# Are some mutations more equal than others?

**DOI:** 10.7554/eLife.87194

**Published:** 2023-04-19

**Authors:** Auden Cote-L’Heureux, Yogesh NK Maithania, Melissa Franco, Konstantin Khrapko

**Affiliations:** 1 Department of Biology, Northeastern University Boston United States

**Keywords:** mitochondrial DNA, somatic mutations, aging, duplex sequencing, Mouse

## Abstract

A large-scale study of mutations in mitochondrial DNA has revealed a subset that do not accumulate with age.

**Related research article** Sanchez-Contreras M, Sweetwyne MT, Tsantilas KA, Whitson JA, Campbell MD, Kohrn BF, Kim HJ, Hipp MJ, Fredrickson J, Nguyen MM, Hurley JB, Marcinek DJ, Rabinovitch PS, Kennedy SR. 2023. The multi-tissue landscape of somatic mtDNA mutations indicates tissue-specific accumulation and removal in aging. *eLife* **12**:e83395. doi: 10.7554/eLife.83395.

Every cell in our body contains hundreds to hundreds of thousands of mitochondria. These organelles are involved in a myriad of functions, most notably respiration (combining oxygen with food to generate energy) and controlled cell death. Mitochondria are the descendants of prokaryotic cells (similar to bacteria) that became part of primordial eukaryotic cells via a process called endosymbiosis: this means that they have their own double-stranded circular DNA (mtDNA), which is similar to the DNA found in bacteria.

The mitochondria within a cell are constantly proliferating and replicating their mtDNA to compensate for cell growth and division, as well as to offset the removal of damaged mitochondria. Because of their respiration activity, mitochondria produce large amounts of reactive molecules, most notably reactive oxygen species, which damage the mtDNA. If a damaged nucleotide in mtDNA remains unrepaired, this may lead to an incorrect nucleotide being inserted during replication, which is then copied onto the new strand during the next round of replication. This results in permanent double-stranded mutations that accumulate with age as mtDNA molecules are continuously damaged and replicated over the course of a person’s lifespan. Consequently, the mutation rates in mtDNA are about a thousand times higher than in nuclear DNA (Khrapko et al., 1997).

In addition to accumulating with age, mutant mtDNAs are also subject to ‘clonal expansion’ within a cell (Coller et al., 2002). This is mostly a stochastic process in which a random mutated mtDNA molecule multiplies and replaces its peers, resulting in the same mutation appearing in most mtDNA molecules in the cell. Clonal expansions are fundamentally important. If enough mitochondria in a cell contain the same mutation, then this mutation will have a phenotypic effect on the entire cell. Moreover, in the female germline, clonal expansions allow mtDNA mutations to take over the egg cell lineage, potentially resulting in the next generation inheriting the mutation.

Most approaches used to analyze mutations in mtDNA suffer a major drawback as they typically involve in vitro DNA replication, such as PCR. This means that damaged nucleotides – which, in vivo, would have likely been repaired prior to replication or excluded from replication – end up getting erroneously copied and eventually converted into artificial double-stranded mutations that are indistinguishable from genuine ones. Now, in eLife, Scott Kennedy and colleagues from the University of Washington – including Monica Sanchez-Contreras and Mariya Sweetwyne as joint first authors – report how they used a technique called duplex sequencing, which excludes these artificial mutations, to study how mtDNA mutations accumulate with age in mice (Sanchez-Contreras et al., 2023).

The team studied eight different tissues – ranging from the kidney, to the brain and the heart – in young and old mice which were 4.5 and 26 months old. This resulted in an unprecedently large dataset consisting of around 80,000 somatic mtDNA mutations, showing that the rate of accumulation and the composition of mutations vary between different tissues. The highest accumulation rate was in the kidney, where mutations reached the level of one in five mtDNA molecules.

In agreement with previous studies, Sanchez-Contreras et al. observed a high proportion of transition mutations, in which purine (adenine and guanine) and pyrimidine (thymine and cytosine) nucleotide bases are only exchanged for other purines or pyrimidines, respectively. An unusually high proportion of transversion mutations – where a purine changes to a pyrimidine, or vice versa – were also detected. However, unlike the transition mutations, these transversions did not clonally expand and did not accumulate with age in any of the tissues studied. Because new transversions are constantly being generated (mostly through damage caused by reactive oxygen species), lack of accumulation with age implies that these mutations are excluded from being propogated in somatic cells.

Intriguingly, another study also found a high proportion of transversion mutations in the mtDNA of mouse egg cells (Arbeithuber et al., 2020), which was surprising given that the proportion of inherited mutations that are transversions is usually very low. This suggests that transversion mutations are excluded from being propagated in germ cells as well.

Next, Sanchez-Contreras et al. treated mice over 24 months of age with one of two drugs that restore mitochondrial function later in life. Compared to control mice, the researchers found that both drugs reduced the fraction of transversion mutations, irrespective of whether they were detrimental or not. This suggests that transversion mutations are not removed by negative selection. Alternatively, transversion mutations are unlikely to be removed via repair mechanisms as the change in base pairs lies across both mtDNA strands and will therefore not be perceived as an error.

So, why do transversions not expand and accumulate with age? And why do transversion and transition mutations behave differently? Furthermore, Sanchez-Contreras et al. point out that despite using a duplex sequencing approach, it is possible that the transversions they detect might have been potentially generated in vitro from real in vivo DNA damage. Although this may be true (see Abascal et al., 2021 for possible mechanisms), the DNA damage present in vivo should have been at least partially converted (also in vivo) into real transversion mutations. So, why did this not result in the accumulation of transversions with age?

We think we might know the answer to some of these questions, and have developed a model which postulates that there are two broad classes of mtDNA molecules (Figure 1): a ‘stem subpopulation’ of actively replicating mtDNAs which are responsible for renewing the mtDNA pool, and a ‘worker subpopulation’ which are located in actively respiring mitochondria. ‘Stem’ mtDNAs reside in mitochondria that respire less and are therefore protected from reactive oxygen species. Consequently, transversions primarily occur on ‘worker’ mtDNA molecules which rarely replicate, which prevents these mutations from being able to clonally expand and accumulate with age. The model is supported by our analysis of HiFi sequencing data collected by Hon et al., 2020, which shows mtDNA molecules falling into high- and low-damaged groups (Cote-L’Heureu et al., 2023), and is also consistent with a functional study showing ‘replicating’ and ‘respiring’ subpopulations of mitochondria in the cell (Döhla et al., 2022).

**Figure 1.**
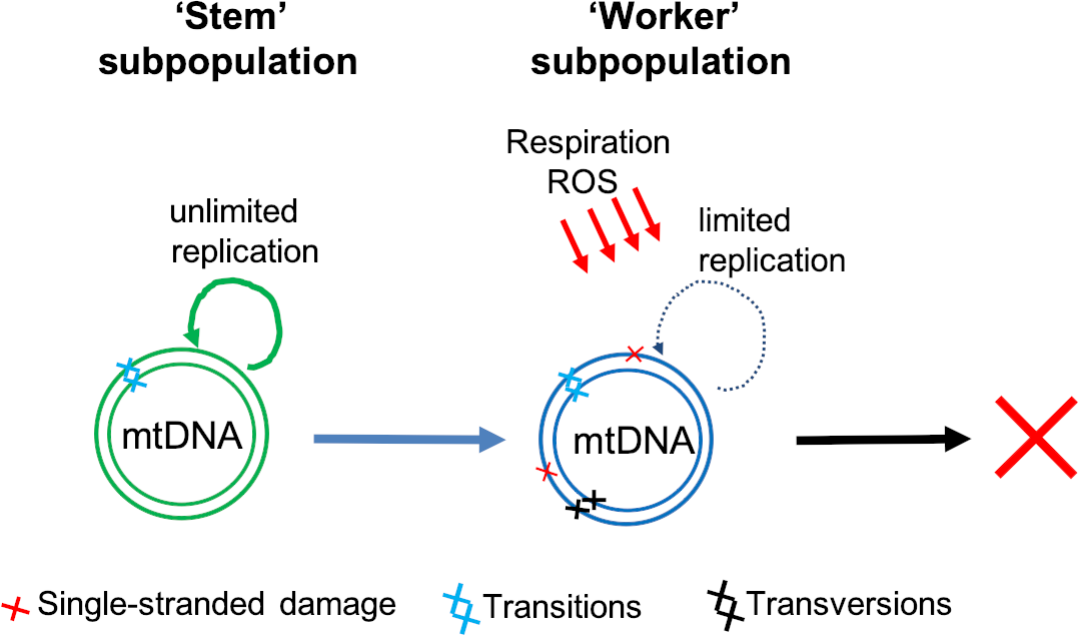
Two classes of mtDNA molecules may explain why transversion mutations do not accumulate with age. Sanchez-Contreras et al. found both transition (light blue crosses) and transversion (black crosses) double-stranded mutations in the mitochondrial DNA (mtDNA) of mice. However, the transversion mutations – which are primarily caused by reactive oxygen species (ROS) – did not accumulate with age. Our own work (Cote-L’Heureu et al., 2023) suggests that cells contain an actively replicating group of mtDNAs – termed the ‘stem’ subpopulation (green) – which are protected from ROS. ‘Stem’ mtDNAs renew themselves and also replenish another group of mtDNAs – termed the ‘worker’ subpopulation (blue) – which are involved in respiration and exposed to higher levels of ROS and therefore acquire more transversion mutations. These mutations, however, do not accumulate because the ‘worker’ mtDNA molecules have a limited replication capacity and are eventually destined for removal (red cross).

In conclusion, the high-fidelity analysis of mtDNA mutations performed by Sanchez-Contreras et al. will become an important resource for future studies. Their data (and similar, more recent work by another group led by Peter Sudmant of Berkeley; Serrano et al., 2023) uncovered an unexpectedly high proportion of transversion mutations in mtDNA molecules, which, unlike transition mutations, did not increase with age.

Work in this field should benefit from the use of single-cell duplex sequencing (Arbeithuber et al., 2020), as well as newer, improved duplex sequencing approaches (Abascal et al., 2021), and long-read mutational analysis (Cote-L’Heureu et al., 2023). These methods could help to test if transversions are real mutations, and how the level of damage varies between mtDNA molecules. Ultimately, this will help researchers to better understand how mtDNA mutations are created, evaded, and inherited, as well as their role in development and aging.
